# A direct-sensing galactose chemoreceptor recently evolved in invasive strains of *Campylobacter jejuni*

**DOI:** 10.1038/ncomms13206

**Published:** 2016-10-20

**Authors:** Christopher J. Day, Rebecca M. King, Lucy K. Shewell, Greg Tram, Tahria Najnin, Lauren E. Hartley-Tassell, Jennifer C. Wilson, Aaron D. Fleetwood, Igor B. Zhulin, Victoria Korolik

**Affiliations:** 1 Institute for Glycomics, Griffith University, Gold Coast Campus, Gold Coast, Queensland QLD 4222, Australia; 2 School of Medical Science, Griffith University, Gold Coast Campus, Gold Coast, Queensland QLD 4222, Australia; 3 Department of Microbiology, University of Tennessee, Knoxville, Tennessee 37996, USA; 4 Computer Science and Mathematics Division, Oak Ridge National Laboratory, Oak Ridge, Tennessee 37861, USA

## Abstract

A rare chemotaxis receptor, Tlp11, has been previously identified in invasive strains of *Campylobacter jejuni,* the most prevalent cause of bacterial gastroenteritis worldwide. Here we use glycan and small-molecule arrays, as well as surface plasmon resonance, to show that Tlp11 specifically interacts with galactose. Tlp11 is required for the chemotactic response of *C. jejuni* to galactose, as shown using wild type, allelic inactivation and addition mutants. The inactivated mutant displays reduced virulence *in vivo*, in a model of chicken colonization. The Tlp11 sensory domain represents the first known sugar-binding dCache_1 domain, which is the most abundant family of extracellular sensors in bacteria. The Tlp11 signalling domain interacts with the chemotaxis scaffolding proteins CheV and CheW, and comparative genomic analysis indicates a likely recent evolutionary origin for Tlp11. We propose to rename Tlp11 as CcrG, Campylobacter ChemoReceptor for Galactose.

## Abstract (web summary)

Some virulent strains of *Campylobacter jejuni* possess a putative chemotaxis receptor, Tlp11, of unknown function. Here the authors show that Tlp11 specifically interacts with galactose and is required for the chemotaxis response of *C. jejuni* to galactose.

## Graphical abstract

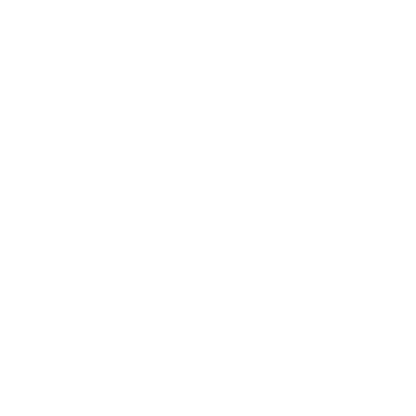

C*ampylobacter jejuni* is now recognized as the leading cause of bacterial gastroenteritis in humans throughout the world, representing a considerable drain on economic and public resources^1^,^2^. The symptoms of campylobacteriosis range from asymptomatic to severe enteritis characterized by fever, severe abdominal cramping and diarrhea with blood and mucus^3^. The bacterium naturally colonizes the gastrointestinal tract of birds and animals, resulting in a commensal relationship, however, consumption of undercooked poultry meat or other food products cross-contaminated during food preparation, give rise to human infections^3^,^4^. Although the molecular details of *C. jejuni* mediated human pathogenesis are not well understood, results of intestinal biopsies of patients and infected primates together with experimental infection of cultured human intestinal epithelial cells, have demonstrated that *C. jejuni* damages and invades host epithelial cells^5^,^6^,^7^,^8^.

In the past, research has been focused on the understanding of virulence factors involved in bacterial adhesion, invasion, and cytotoxin production. Some of the known putative virulence factors include fibronectin-binding protein (CadF)^9^, cytolethal distending toxin^10^, campylobacter invasion antigens (Cia proteins)^11^, chemotaxis mediated motility^12^, lipoprotein (JlpA)^13^, a phase-variable capsule^14^ and the pVir plasmid^15^. While *C. jejuni* is known to colonize the intestinal mucus, particularly within the cecal crypts, and is able to overcome the physical and immunological barrier posed by the intestinal mucus layer in order to establish an infection in humans^16^, not much is known on how *C. jejuni* targets the mucus lining of the small and large intestine of both birds and mammals. The motility and corkscrew morphology of *C. jejuni* is thought to allow it to penetrate the mucus layer^17^ and mucin and L-fucose are known chemoattractants for *C. jejuni*^12^. More recently, the interactions of *C. jejuni* with mucus and the mucin glycoproteins, the main component of mucus, have been investigated. *C. jejuni* was always considered an asaccharolytic organism, however, studies have revealed that certain strains of *C. jejuni* can uptake and metabolize the sugar L-fucose, one of the sugars present on mucin glycoproteins^18^,^19^.

Motility and chemotaxis have been shown to be critical for *C. jejuni* infection and colonization of its hosts^20^. Chemotaxis receptors, also referred to as chemoreceptors, are important in the activation of the chemotaxis signal transduction cascade. Chemotaxis receptors are called methyl-accepting chemotaxis proteins, or transducer-like proteins, and detect various stimuli in the environment and transduce signals into the cytoplasm modulating the rotational directionality of the flagellar motor^21^. Although tens of thousands of chemoreceptor sequences are available in current databases, ligand-binding specificity is known for only a small number of these proteins. The majority of *C. jejuni* laboratory strains, such as *C. jejuni* subsp. *jejuni* strains NCTC 11168 (ATCC 700819) and ATCC 81116, have 10 chemoreceptors (data from the MiST database^22^). Six of these chemoreceptors belong to the class I membrane topology group^23^, where two transmembrane regions demarcate a periplasmic ligand-binding domain. Recently, we and others have identified directly binding ligands for three such *C. jejuni* chemoreceptors: CcaA (Tlp1), CcmL (Tlp3) and Tlp7 (refs 24, 25, 26). The receptor specificities ranged from a single ligand (aspartate for CcaA) to multiple different and distinct ligands (10 in total for CcmL)^24^,^25^,^26^. Previous screening of a range of human, avian and newly obtained clinical isolates showed the presence of different subsets of *tlp* genes coding for class I (group A) chemoreceptors in different strains^27^. Tlp11 is a recently identified chemoreceptor present in strains that were known to lead to disease requiring hospitalization^27^.

In this study, we characterize the Tlp11 chemosensory receptor (which we propose to rename CcrG), identifying the receptor specific ligand, galactose, and showing its recent evolutionary origins and occurrence in only a few, virulent strains. We also study the role for this receptor in adherence to host cells and colonization of chickens.

## Results

### Ligand-binding specificity of Tlp11

The Tlp11 sensory receptor was found in approximately 11% (5/44) of *C. jejuni* isolates from humans and chickens via PCR analysis using *tlp11* gene specific primers (Supplementary Table 1). The five strains carrying *tlp11* were invasive or highly invasive. However, *tlp11* could not be found in six *C. jejuni* strains previously described as hyperinvasive^28^.

To determine ligand specificity of Tlp11, the periplasmic sensory domain of this gene, Tlp11^peri^, was cloned distal to a His-tag in an expression vector pET-19b (plasmids described in Supplementary Table 2). The recombinant protein was expressed and purified. Ligand-binding specificity of the His-tagged Tlp11^peri^ was assessed using amino acid, glycan and small molecule arrays. No binding was observed on the chemotaxis ligand array (for the full list of amino acids and salts see Methods), but binding was noted to terminal galactose structures (Table 1, for the full range of glycans tested see Supplementary Table 3). The binding was observed to any free terminal galactose regardless of underlying linkage, however, galactose with a further distal sugar residue bound, such as a sialic acid or fucose, was not recognized (Table 1). Furthermore, no terminal *N*-acetylgalactosamine structures were recognized. Surface plasmon resonance of free galactose, glucose and ribose was performed. Glucose and ribose were included to confirm the absence of binding to these glycans since in *Escherichia coli* the galactose receptor also recognizes glucose and ribose. The disassociation equilibrium constant (*K*_D_) of Tlp11^peri^ for galactose was determined to be 17 μM (±5.7 μM). No binding to glucose or ribose was detected (Supplementary Fig. 1). Saturation transfer difference (STD) NMR analyses were also undertaken as previously described^24^ to ensure the specificity of the Tlp11-galactose binding. Tlp11^peri^ binding to galactose, but not glucose or aspartate, which were included as non-binding controls, was detected (Supplementary Fig. 2). As for the glycan array, surface plasmon resonance and STD-NMR identified galactose as the only ligand for Tlp11. Therefore, hereafter we refer to this receptor as CcrG—Campylobacter ChemoReceptor for Galactose

### CcrG origins and phyletic distribution

BLAST analysis of the full length *ccrG* gene showed 95–98% identity at the nucleotide level (100% identity in the periplasmic domain region) among *ccrG* sequences in all strains that encode this receptor. The search for CcrG orthologs was conducted not only with the full length sequence as a query, but also with the sequences corresponding to the periplasmic ligand-binding domain (CcrG^peri^), and the cytoplasmic signalling domain as separate queries. Full length, CcrG^peri^ and the cytoplasmic signalling domain queries all resulted in nearly identical distributions of BLAST hits thus unambiguously identifying CcrG orthologs only in a small subset of strains: three other *C. jejuni* strains, two strains of *Campylobacter coli* and one strain of *Campylobacter upsaliensis* (Supplementary Fig. 3). The closest relative of CcrG is the chemoreceptor annotated as TlpA in *C. coli*, which is present in many *Campylobacter* strains, including the widely used laboratory strain 81116, as well as in *Helicobacter* strains (Supplementary Fig. 3). Thus, CcrG appears to be the TlpA paralog, which arose through recent *tlpA* duplication. Because the function of TlpA is unknown, we have compared CcrG with other class I chemoreceptors in *C. jejuni*, whose function is known—the aspartate chemoreceptor CcaA^24^ and the multi-ligand chemoreceptor CcmL^25^. Interestingly, the CcrG periplasmic ligand-binding domain (residues 32 to 332 in CcrG) shares 35% identity with CcaA and 20% identity with CcmL, however, the cytoplasmic signalling domain (residues 376 to 707 in CcrG) shares 90% identity with CcmL and only 64% identity with CcaA. The CcrG/TlpA type of chemoreceptors may have originated through a domain swap at some point in the evolution of the *Campylobacter*/*Helicobacter* clade. Because of its recent appearance and low sequence identity to known chemoreceptors, we have carried out a detailed sequence analysis of the CcrG ligand-binding region.

### Sequence analysis of the CcrG ligand-binding region

**Figure 1:**
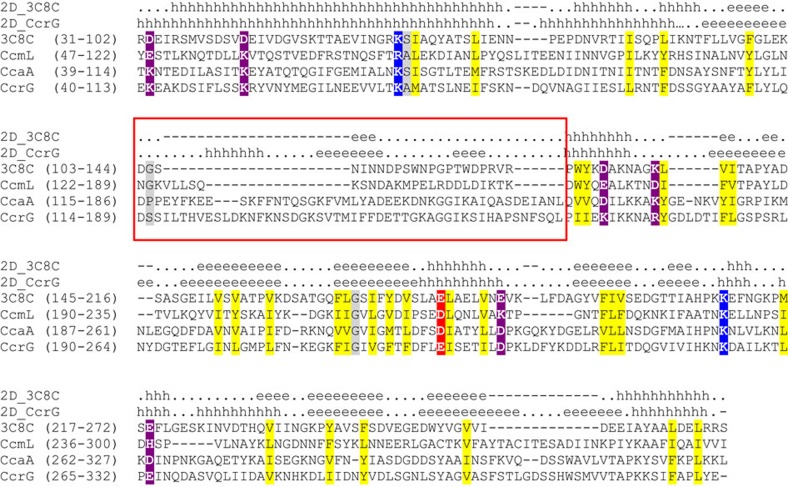
Periplasmic regions of selected chemoreceptors. Multiple sequence alignment of the periplasmic region from the *Vibrio cholerae* Mcp37 chemoreceptor (PDB: 3C8C) with those from *C. jejuni* chemoreceptors. Known (3C8C) and predicted (CcrG) secondary structure elements are shown above the alignment: h, alpha-helix; e, beta strand. Conserved residues are highlighted in yellow (hydrophobic), purple (charged), blue (positively charged), red (negatively charged) and grey (small). Regions of poor conservation are shown within a red box. Locus tag numbers: 3C8C, VCA0923; CcaA, Cj1506c; CcmL, Cj1564; CcrG,N135_00253.

Sequence-based searches against the Pfam^29^ and CDD^30^ domain databases revealed no known domains in the CcrG periplasmic region (residues 32 to 332); however, similar searches with the corresponding region from its closest homologues (for example, YP_008623181 and YP_008746227) did reveal the dCache_1 domain (Pfam accession number PF02743), suggesting its presence in CcrG. We then used a more sensitive, profile-profile search against the same domain databases using the HHPred tool^31^, which has confidently (>99.7% probability) identified the dCache_1 domain not only in all CcrG orthologs, but also in all homologous chemoreceptors, including CcmL and CcaA. HHPred searches against protein structures from the PDB database identified the structure of the ligand-binding region of the *Vibrio cholerae* chemoreceptor (PDB code 3C8C) as the most closely related to *C. jejuni* chemoreceptors. Consequently, we have constructed a multiple sequence alignment of these regions to reveal potential structural similarities and differences between them. While the overall predicted structure of CcmL follows that of the known 3C8C structure, both CcrG and CcaA have distinct deviations, especially in the N-terminal region corresponding to the membrane-distal subdomain (Fig. 1), which is the site for ligand binding in 3C8C. In order to survey all known ligands for dCache_1 domains, we have searched the National Center for Biotechnology Information (NCBI) PubMed records for gene/protein identifiers for all proteins in the NCBI protein database that contain dCache_1 domains. Table 2 contains information on chemoreceptors and histidine kinases, where ligands were shown to be directly bound by extracellular regions containing the dCache_1 domain. The results show that dCache_1 domains bind amino acids and to a lesser extent organic acids. We have found no reports of a sugar being a main ligand for a dCache_1 domain. We further investigated the ligand-binding regions of dCache_1 containing Group A chemoreceptors through phylogenetic methods. The results of our phylogenetic reconstruction (Supplementary Fig. 3) show that periplasmic regions from dCache_1 Group A chemoreceptors conform to one of two types in *C. jejuni*, *C. coli* and *C. upsaliensis*: CcmL-like receptors (Tlp2, Tlp3 (CcmL) and Tlp4) and CcaA-like receptors (which comprise Tlp1 (CcaA) and Tlp11 (CcrG)). Furthermore, and most striking, the residues in these receptors that correspond to ligand-binding residues identified in 3C8C are differently conserved (either by identity and/or biochemical properties) between the two types (Fig. 1).

Interrogation of 169 *C. jejuni* genomes showed that, while *C. jejuni* has the ability to synthesize galactose from other metabolites, no galactose catabolic genes could be found in any of the *C. jejuni* sequences. Additionally, none of the CcrG containing strains had any genes that had homology to proteins for uptake of galactose or other sugars when using the common amino acid domains as the query sequence.

### Effects of CcrG on autoagglutination

**Figure 2:**
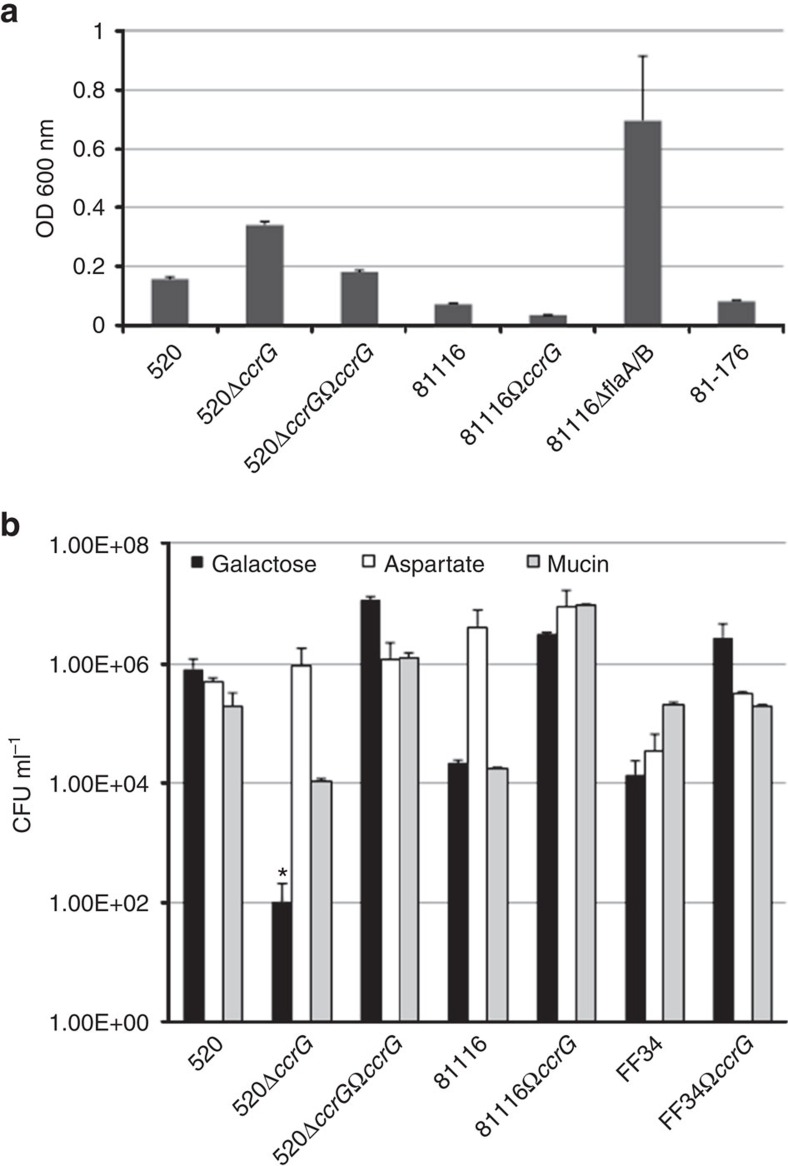
Effects of CcrG on autoagglutination and chemotaxis. Autoagglutination and chemotaxis assays of wild-type *C. jejuni* strains 520 and 81116, mutant (520*ΔccrG*), complemented mutant (520Δ*ccrG*Ω*ccrG*) and knock-in (81116Ω*ccrG*). (**a**) Autoagglutination was tested for each of the strains with *C. jejuni* strain 81–176 used as a positive control and *C. jejuni* non-motile mutants (81116Δ*flaA*/*flaB*) used as a negative control. Data is displayed as the OD_600nm_ of the supernatant with all strains starting from the same OD_600nm_. The change observed at the OD_600nm_ indicates a change in autoagglutination. (**b**) Chemotaxis assays were performed using aspartate and mucin as positive motility controls with galactose as the test compound. The *C. jejuni* non-motile mutant (81116Δ*flaA*/*flaB*) was used as the negative control. No movement was observed to galactose and a no-treatment control. Data is from three replicate experiments. The asterisk (*) indicates significant difference compared with wild-type strain (*P*<0.05; *t*-test). s.d. errors are shown as bars above the mean.

The presence of *ccrG* had significant effects on autoagglutination of the harbouring strain. In *C. jejuni* strain 520, the insertionally inactivated 520Δ*ccrG*::Km^R^ showed, on average, a 2.5-fold reduction in agglutination compared with the wild-type 520 strain, while the allelic insertion 81116Ω*ccrG* showed a twofold increase in agglutination compared with the wild-type 81116. The complemented mutant strain 520Δ*ccrG*::Km^R^Ω*ccrG*::Cat^R^ (abbreviated to 520Δ*ccrG*Ω*ccrG* from here onwards) showed full complementation of the mutant phenotype (*P*=0.09; *t*-test) compared with wild-type 520. This indicates that CcrG positively influences *C. jejuni* autoagglutination (Fig. 2a). This is not the first Class I chemoreceptor to have a role in bacterial agglutination, as CcmL was found to negatively influence autoagglutination of *C. jejuni*.

### Chemotaxis of *C. jejuni* isogenic strains with and without CcrG

Chemotactic responses of the isogenic strains, with and without CcrG, to galactose were determined. Assays were performed using the *C. jejuni* 520 wild-type strain, insertionally inactivated 520Δ*ccrG*::Km^R^ isogenic mutant, the complemented mutant strain 520Δ*ccrG*Ω*ccrG*, the wild-type 81116 strain and its allelic insertion isogenic strain 81116Ω*ccrG*, as well an invasive human *C. jejuni* strain FF34 and its allelic insertion isogenic strain FF34Ω*ccrG*.

The *C. jejuni* 520Δ*ccrG*::Km^R^ isogenic mutant had significantly reduced chemotactic motility towards galactose (by three orders of magnitude; *P*<0.05; *t*-test), as compared with the wild-type *C. jejuni* 520 and the complemented mutant strain 520Δ*ccrG*Ω*ccrG* (Fig. 2b). To ensure that the change in chemotaxis towards galactose was not due to a motility defect, we measured chemotaxis towards positive chemotactic motility controls, mucin and aspartate; we found that chemotaxis towards these positive controls was not affected by deletion of CcrG. A FlaAB non-motile mutant was used as a negative control. It is interesting to note that when the *ccrG* allele was added to the genomes of *C. jejuni* 81116 and FF34 that normally lack this receptor, the chemotaxis towards galactose was increased by two orders of magnitude (Fig. 2b, *P*<0.05; *t*-test) demonstrating that movement towards galactose was CcrG dependent.

### *In vitro* adherence and invasion assays

**Figure 3:**
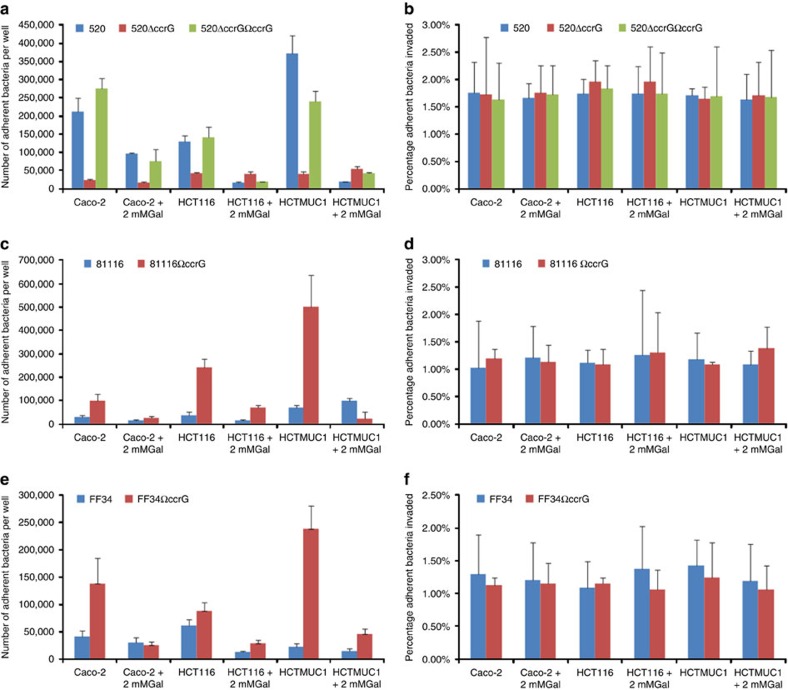
Adhesion and invasion analysis. Adhesion (**a**, **c**, **e**) and invasion (**b**, **d**, **f**) analysis of Caco2, HCT116 and HCT116Ω*muc1* cell lines on *C. jejuni* 520 wild-type, 520Δ*ccrG*::Km^R^, 520Δ*ccrG*Ω*ccrG* (**a** and **b**), 81116 wild-type, 81116Ω*ccrG* (**c**, **d**), FF34 wild-type and FF34Ω*ccrG* (**e**, **f**) strains. Adhesion and invasion analyses are presented as the mean of invasion from three replicate experiments and standard deviation errors are shown as bars above the mean (*P*<0.05; *t*-test).

To assess the biological significance of CcrG in *C. jejuni* strains, we compared their adherence to and invasion of polarized Caco-2 cells and the human colorectal cancer cell line HCT116. We tested the following strains: 520Δ*ccrG*::Km^R^ (isogenic inactivation) 520Δ*ccrG*Ω*ccrG* (complemented isogenic inactivation), 81116Ω*ccrG* and FF34Ω*ccrG* (allelic addition) mutants, in comparison with wild-type strains 520, 81116 and FF34. Adherence and invasion into HCT116 cells with and without over-expression of MUC1 surface mucin (HCT116Ω*muc1* (ref. 32), rich in galactose, fucose and sialic acid glycosylation was also assessed. Adherence and invasion assays were also performed in competition with free galactose. Expression of CcrG in 520 wild-type, 520Δc*crG*Ω*ccrG*, 81116Ω*ccrG* and FF34Ω*ccrG* strains resulted in higher adherence compared with *C. jejuni* strains not expressing CcrG in all cell types tested (*P*<0.05; *t*-test; Fig. 3). MUC1 over-expression in HCT116 cells also resulted in higher adherence of all strains expressing CcrG, when compared with CcrG expressing strains adhering to regular HCT116 cells (*P*<0.05; *t*-test Fig. 3), indicating that CcrG plays a role in sensing components of mucin. Addition of 2 mM galactose to the media during adherence assays nullified the increased adherence of CcrG expressing strains, while not significantly affecting the adherence of *C. jejuni* strains not expressing CcrG. CcrG expression was not linked to any significant change in invasion of Caco-2 cells, HCT116 cells or HCT116 cells over-expressing MUC1 (Fig. 3b,d,f).

### Colonization potential of *C. jejuni* wild-type and mutant strains

Approximately 48 h after hatching, chickens were orally infected with different doses of *C. jejuni* 520 wild type, 520Δ*ccrG*::Km^R^, 81116 wild type and 81116Ω*ccrG* strains. At 5 days post infection, different strains of *C. jejuni* in the caeca were enumerated. The 520 wild type and 520Δ*ccrG*::Km^R^ were able to colonize all chickens with the inocula of 1 × 10^8^, 1 × 10^6^ and 1 × 10^4^ colony-forming unit (c.f.u). The bacterial loads of the 520 wild type in the caeca ranged from 9.2 × 10^7^ to 7.9 × 10^7^ c.f.u. per gram of caecal content while bacterial loads of the 520Δ*ccrG*::Km^R^ ranged from 4.4 × 10^6^ to 7.0 × 10^5^ c.f.u. per gram of caecal content. 520Δ*ccrG*::Km^R^ displayed a significant, 10- to 100-fold, reduction in colonization when compared with that of the 520 wild type (*P*<0.05; *t*-test). No difference was observed when the colonization potential of chickens by *C. jejuni* 81116 wild type and 81116Ω*ccrG* mutant was tested.

### The CcrG signalling domain interacts with CheW and CheV

To further confirm the role of CcrG as a chemoreceptor in *C. jejuni* 520, a yeast two-hybrid system was used to analyse interactions between the predicted cytoplasmic signalling domain of CcrG and the homologues of the scaffolding proteins of the chemotaxis signalling pathway in *C. jejuni*, CheV and CheW, as previously described^24^. Residues 562–707 of CcrG (CcrG^sig^), encompassing the region homologous to the highly conserved bacterial methyl-accepting chemotaxis protein signalling domain, were interrogated for protein–protein interactions (Supplementary Table 4). This region is almost identical to the predicted cytoplasmic signalling domain of the *C. jejuni* Group A transducer-like proteins of *C. jejuni* 11168, Tlp2 (Cj0144), CcmL (Cj1564) and Tlp4 (Cj0262). A medium strength interaction was detected between CcrG^sig^ and CheV (AD-CcrG^sig^ and BD-CheV). This interaction was also detected in the reciprocal combination of fusion proteins (BD-CcrG^sig^ and AD-CheV). CcrG^sig^ was found to interact with the CheW-like domain of CheV (CheV^dW^) (AD-CcrG^sig^ and BD-CheV^dW^). Similarly, CcrG^sig^ and CheW were found to interact in the combination of fusion proteins BD-CcrG^sig^ and AD-CheW (Supplementary Table 4). The CcrG signalling domain was also found to interact with itself suggesting dimerization of this chemoreceptor.

## Discussion

In this study, we have shown that CcrG (Tlp11) acts as a direct-sensing galactose chemoreceptor for *C. jejuni* 520. Mutation of the *C. jejuni ccrG* gene altered phenotypic characteristics of the bacteria including chemotactic motility and autoagglutination behaviour, suggesting a role for this chemoreceptor in the interaction of *C. jejuni* with its hosts. We demonstrated that the sensory domain of the *C. jejuni* CcrG chemoreceptor has binding specificity to galactose with the *K*_D_ of CcrG to galactose in a biologically relevant range (17 μM), similar to those observed for other *C. jejuni* sensory receptors^24^,^25^. Increased chemotaxis towards galactose of allelic addition strains, 81116Ω*ccrG* and FF34Ω*ccrG,* further confirmed that the chemotaxis response to galactose was affected by CcrG. Therefore, we propose to name this receptor CcrG.

In a previous study by Hugdahl *et al*.^12^, chemotaxis of *C. jejuni* to galactose was not detected. We think it is likely that the strains used in that study did not contain CcrG; so far this receptor has been identified in only a few highly virulent, closely related strains^33^. This fact strongly suggests recent evolutionary origins of CcrG, which is further corroborated by protein sequence analysis.

Bacterial chemoreceptors have unevenly conserved domains. The cytoplasmic signalling domains show extremely high conservation levels due to evolutionary pressure to maintain multiple protein-protein interactions^34^. Conversely, the ligand-binding (sensory) domains detect numerous signals^35^ and are highly variable in sequence, as each chemoreceptor adapts to sense specific physicochemical queues, often after gene duplication^35^. In the case of CcrG, its ligand-binding and signalling domains are most similar to corresponding regions in two different chemoreceptors, suggesting a recent domain swap. Extreme sequence conservation of the CcrG signalling domain and its ability to bind CheW and CheV scaffolding proteins suggest that it is an integral part of the chemosensory signalling array identified in *C. jejuni*^36^.

In *C. jejuni*, CcrG appears to bind galactose directly and does not bind either glucose or ribose. This is in contrast to the model organism, *E. coli,* where the galactose/glucose and ribose chemotaxis is mediated by periplasmic sugar-binding proteins interacting with the Trg chemoreceptor^37^. Furthermore, the galactose/ribose binding protein in *E. coli* belongs to a different structural fold than the dCache_1 domain of the CcrG chemoreceptor further highlighting the novel mechanism for galactose sensing in *C. jejuni* chemotaxis. dCache_1 domains comprise the largest group of extracellular sensors in bacteria^36^; however, to our knowledge, CcrG is the first dCache_1-containing protein to directly bind a sugar as the main ligand. Ligands directly binding to dCache_1 domains in other chemoreceptors and sensory histidine kinases from various bacteria include primarily amino and organic acids (Table 2).

It is interesting to note that *C. jejuni* strain 81116 is chemotactic towards galactose, indicating that there is another receptor or a receptor-periplasmic binding protein pair that may be responsible for sensing galactose in the *C. jejuni* strains lacking the CcrG receptor. The introduction of the eleventh receptor, CcrG, was able to enhance the chemotactic response of the *C. jejuni* 81116Ω*ccrG* isogenic strain by several orders of magnitude. Moreover, enhanced galactose sensing may contribute, among other factors, to enhanced virulence of CcrG carrying strains, despite the fact that the presence of CcrG is not correlated with a hyperinvasive phenotype. Additionally, the CcrG encoding *C. jejuni* cells have significantly increased adherence to human cell lines expressing the surface mucin MUC1. The sensing of MUC1 by CcrG could be linked to the increase in adherence as this can be completely blocked by free galactose in the media. It is possible that CcrG expressing bacteria sense galactose terminating glycans, which are abundant on MUC1. Consequently, CcrG may have a role in directly sensing host cell surface glycans, thereby potentially enhancing the ability of CcrG expressing strains to cause disease.

Further characterization of the ligands of *C. jejuni* chemosensory proteins will contribute to understanding not only the chemotaxis signalling pathways involved in colonization and invasion, but also the importance of external signals in the survival and pathogenesis of this organism. The identification of a unique Cache domain-containing chemoreceptor, directly sensing galactose, has implications not only for *C. jejuni* chemotaxis but for other bacteria, many of which utilize Cache domains as environmental sensors^36^.

## Methods

### Bacterial strains and growth conditions

Strains and plasmids used in this study are described in Supplementary Table 2. *C. jejuni* 81116 and 11168 were kindly provided by Diane Newell, Veterinary Laboratories Agency, UK. Hyperinvasive strains of *C. jejuni* were kindly provided by Georgina Manning, Nottingham Trent University, UK. *C. jejuni* and *E. coli* strains were grown as described previously^24^.

### Preparation of CcrG periplasmic sensory domain

Screening of *C. jejuni* strains for CcrG and PCR for cloning of the DNA sequence encoding the CcrG periplasmic sensory domain, CcrG^peri^, into a protein expression vector pET-19b (Novagen) were performed using primers for CcrG-periplasmic region Tlp11mid F: CTC TGA TGG CAA AAG TGT AAC and Tlp11mid R: CTC TTC AGA TTG AGC GAT AAC, previously published in Day *et al*^27^. The CcrG^peri^ protein was purified and verified as described for CcaA in Hartley-Tassell *et al*.^24^.

### Identification of protein ligand interactions for CcrG

Glycan and small molecule arrays were performed as described previously^24^,^25^. Briefly, 1 μg of protein precomplexed with anti-His antibody (Cell Signaling) and rabbit anti-mouse/goat anti-rabbit Alexa 555 IgG (Thermo Scientific) in a molar ratio of 1:0.5:0.25 was incubated on the array for 30 min in phosphate buffered saline (Thermo Scientific). Arrays were scanned and analysed using ScanArray Express (Perkin Elmer). Small molecule array contained: alanine, arginine, asparagine, aspartate, cysteine, fumaric acid, glucosamine, glutamic acid, glutamine, histidine, isoleucine, leucine, lysine, malic acid, methionine, phenylalanine, proline, purine, serine, succinic acid, thiamine, threonine, tryptophan, tyrosine, valine and α-ketoglutarate. Biacore and STD-NMR analysis were performed as described in Rahman *et al*.^25^ with galactose, ribose and glucose used in place of the amino acids in the same concentration range.

### Insertional inactivation of ccrG

*C. jejuni* 520 *ccrG* coding region was amplified using forward 5′-ATG AAT TTT CGT TCT CTA AAT TTA AG-3′ and reverse 5′-TTA ATG ATT CTC TTC CTT CTT AAC-3′ primers. The PCR product was cloned into pGEMT-Easy (Promega). The coding region was interrupted by insertion of a non-polar 1.1-kb kanamycin resistance cassette from a recombinant plasmid pMW10 (ref. 38) into a unique *Bgl*II site using *Bgl*II flanked primers (forward 5′-AGA TCT GCT CGG AAT TAA CCC TCA C-3′and reverse 5′-AGA TCT CTG TTT TCT GGT ATT TAA G-3′). The isogenic *ccrG* mutant, 520Δ*ccrG*::Km^R^, was constructed by electrotransformation of *C. jejuni* strain 520 and verified as previously described^39^. The 520Δ*ccrG*::Km^R^ mutant was complemented by the insertion of *C. jejuni* 520 *ccrG* coding region, which was amplified using the forward and reverse primers flanked with *Bsm*BI sites shown above. The PCR product was first cloned into pGEMT-Easy (Promega) and subcloned into pC46 (ref. 40). The *ccrG* containing pC46 vector was cloned into 520Δ*ccrG*::Km^R^ using natural transformation and plated onto plates containing 50 μg ml^−1^ kanamycin and 10 μg ml^-1^ chloramphenicol. The complemented strain 520Δ*ccrG*Ω*ccrG* was tested by PCR to confirm the presence of the uninterrupted *ccrG* gene fragment and the chloramphenicol resistance gene. There was no growth rate difference between *C. jejuni* wild-type 520, 520Δ*ccrG*::Km^R^ or 520Δ*ccrG*Ω*ccrG* (Supplementary Fig. 4a; *P*>0.2; *t*-test).

### Construction of allelic addition isogenic mutants

To create the heterologous gene expression mutant, the entire coding region of the 520 *ccrG* gene, including the start and stop codons, was ligated directly into pBF6A (pBluscript*ΩC.jejuni::flaA::flaB::*Km^R^Amp^R^; Benjamin Fry, RMIT University, Melbourne, Australia, unpublished) into the non-essential *flaB* gene in the same orientation and electroporated into *C. jejuni* strains 81116 and FF34, to add a *ccrG* allele to the genome. A control motility mutant carrying the Km^R^ cassette within the *C. jejuni* strain 81116 *flaB* gene was similarly constructed and normal motility verified. No difference in growth rate was observed between *C. jejuni* 81116 wild type and 81116Ω*ccrG* strains (Supplementary Fig. 4b; *P*=0.85; *t*-test).

### Verification of expression of ccrG alleles

Expression levels of *ccrG* were tested in 520Δ*ccrG*::Km^R^ (isogenic inactivation), FF34Ω*ccrG* and 81116Ω*ccrG* (allelic addition) compared with wild-type controls by real-time PCR using primers Tlp11mid F and R described above. *C. jejuni* 520Δ*ccrG*::Km^R^ was found to express *ccrG* 1.93(±0.34) fold lower than 520 wild-type control. 520Δ*ccrG*Ω*ccrG* was found to express *ccrG* 3.22 (±0.42) fold above the 520 wild-type control. While the FF34Ω*ccrG* and 81116Ω*ccrG* expression levels were 2.47 (±0.68) fold and 2.63 (±0.29) fold higher than 520 wild-type control, respectively. *C. jejuni* 81116 and FF34 wild-type strains were confirmed to lack *ccrG* expression.

### Adherence and invasion assays of culture cell lines Caco-2 and HCT116

Caco-2, HCT116 and HCT116 overexpressing MUC1 adherence and invasion assays were performed with and without centrifugation as previously described^24^,^32^,^41^. Briefly, cells were grown to confluence in a 24-well plate and adherence/invasion assays were performed with a starting inoculum of ∼10^8^ bacteria per well. Galactose competition was performed by pre-incubating *C. jejuni* with 2 mM galactose and keeping galactose at 2 mM throughout the adherence assay.

### Soft agar motility assay and nutrient depleted chemotaxis assay

Motility and chemotaxis assays were performed as previously described^25^. Briefly, 6 mm plugs were removed from a 0.5% agar (in H_2_O) plate and replaced with 0.5% agar with 2 mM of amino acid, buffer or mucin. The plates were overlaid with 0.1% agar in H_2_O (no nutritional supplements) and left for 2 h to allow for diffusion. A 24 h culture of *C. jejuni* strains, grown at 42 °C, was sub-cultured in Brucella broth for a further 18 h with shaking at 50 r.p.m. Cells were collected by centrifugation, resuspended in saline and 10^8^ c.f.u. of *C. jejuni* in a 100 μl drop were then inoculated in the center of the petri dish. Viable bacteria that have migrated to amino acid plugs after 4 h at 37 °C, were enumerated.

### Analysis of the isogenic ccrG mutant in chicken colonization

The 2-day model for chicken colonization was used^42^ to assay the effects of isogenic *ccrG* mutation on bacterial colonization potential as previously described^24^,^25^. Briefly, Groups of 8 to 10 two-day old chicks in isolators were inoculated orally with 10^6^ c.f.u. of *C. jejuni* strain 520 or isogenic mutants of this strain. Colonization was monitored daily by culture from cloacal swabs. Chickens were euthanized 5 days following inoculation, and the level of colonization was enumerated.

### Yeast two-hybrid analysis

Yeast two-hybrid analysis of protein interactions was performed as described previously^24^. Interactions involving BD-CcrG^sig^ were analysed on intermediate stringency containing 1 mM 3-AT, as this media was found to suppress the autonomous activation of reporter gene expression exhibited by this fusion protein.

### Bioinformatics

BLAST searches^43^ against the RefSeq database at NCBI were carried out with default parameters. Protein domain architectures were obtained from the Pfam^29^ and Conserved Domain^30^ databases. Sensitive profile-profile searches for domain identification were carried out using HHpred^31^. Multiple sequence alignments were constructed using LINS-I algorithm from the MAFFT v7 program^44^. Maximum-likelihood phylogenetic trees were constructed using the MEGA6 package^45^.

### Data availability

The data that support the findings of this study are included in this published article and its Supplementary Information files, or are available from the corresponding author upon request.

## Additional information

**How to cite this article:** Day, C. J. *et al*. A direct-sensing galactose chemoreceptor recently evolved in invasive strains of *Campylobacter jejuni*. *Nat. Commun.* **7,** 13206 doi: 10.1038/ncomms13206 (2016).

## Supplementary Material

Supplementary Information Supplementary Figures 1 - 4, Supplementary Tables 1 - 4 and Supplementary References

## Figures and Tables

**Table 1:** Glycan structures recognized by CcrG.

**Glycan**	**Structure**
1A	Galβ1-3Glc*N*Ac
1B	Galβ1-4Glc*N*Ac
1C	Galβ1-4Gal
1D	Galβ1-6Glc*N*Ac
1E	Galβ1-3Gal*N*Ac
1F	Galβ1-3Gal*N*Acβ1-4Galβ1-4Glc
1G	Galβ1-3Glc*N*Acβ1-3Galβ1-4Glc
1H	Galβ1-4Glc*N*Acβ1-3Galβ1-4Glc
1I	Galβ1-4Glc*N*Acβ1-6(Galβ1-4Glc*N*Acβ1-3)Galβ1-4Glc
1J	Galβ1-4Glc*N*Acβ1-6(Galβ1-3Glc*N*Acβ1-3)Galβ1-4Glc
1K	Galα1-4Galβ1-4Glc
1M	Galβ1-3Gal*N*Acα1-*O*-Ser
1N	Galα1-3Gal
1O	Galα1-3Galβ1-4Glc*N*Ac
1P	Galα1-3Galβ1-4Glc
2A	Galα1-3Galβ1-4Galα1-3Gal
2B	Galβ1-6Gal
2E	Galα1-4Galβ1-4Glc*N*Ac
2G	Galβ1-3Glc*N*Acβ1-3Galβ1-4Glc*N*Acβ1-6(Galβ1-3Glc*N*Acβ1-3)Galβ1-4Glc
2H	Galβ1-3Glc*N*Acβ1-3Galβ1-4Glc*N*Acβ1-3Galβ1-4Glc
7B	Galβ1-3(Fucα1-4)Glc*N*Acβ1-3Galβ1-4Glc
7C	Galβ1-4(Fucα1-3)Glc*N*Acβ1-3Galβ1-4Glc
7D	Fucα1-2Galβ1-3(Fucα1-4)Glc*N*Acβ1-3Galβ1-4Glc
7E	Galβ1-3(Fucα1-4)Glc*N*Acβ1-3Galβ1-4(Fucα1-3)Glc
7H	Galβ1-4(Fucα1-3)Glc
7I	Galβ1-4(Fucα1-3)Glc*N*Ac
7J	Galβ1-3(Fucα1-4)Glc*N*Ac
7M	Galβ1-3(Fucα1-2)Gal
8C	Galβ1-3Glc*N*Acβ1-3Galβ1-4(Fucα1-3)Glc*N*Acβ1-3Galβ1-4Glc

**Table 2:** Ligands directly binding to dCache_1 domain-containing periplasmic regions of bacterial receptor proteins.

Receptor*	**Ligands**
McpB, *Bacillus subtilis* (BSU31260) (ref. 46)	Asparagine, aspartate, glutamine, histidine
McpC, *Bacillus subtilis* (BSU13950) (ref. 47)	Proline, threonine, glycine, serine, valine, alanine, tyrosine, isoleucine, tryptophan, phenylalanine, leucine, histidine
KinD, *Bacillus subtilis* (BSU13660) (ref. 48)	Pyruvate, propionate, butyrate
PctA, *Pseudomonas aeruginosa* (PA4309) (ref. 49)	Arginine, lysine, tyrosine, tryptophan, phenylalanine, alanine, valine, isoleucine, leucine, methionine, asparagine, serine, cysteine, threonine, histidine, proline, glycine
PctB, *Pseudomonas aeruginosa* (PA4310) (ref. 49)	Arginine, lysine, alanine, methionine, glutamine
PctC, *Pseudomonas aeruginosa* (PA4307) (ref. 49)	Histidine, proline, gamma-aminobutyrate (GABA)
Mlp24, *Vibrio cholera* (VC2161) (ref. 50)	Serine, arginine, asparagine, proline
Mlp37, *Vibrio cholerae* (VCA0923) PDB:3C8C	Alanine
CcaA, *Campylobacter jejuni* (Cj1506c) (ref. 24)	Aspartate
CcmL, *Campylobacter jejuni* (Cj1564) (ref. 25)	Isoleucine, lysine, arginine, aspartate, glucosamine, succinate, malate, fumarate, α-ketoglutarate, purine, thiamine,
McpU, *Sinorhizobium meliloti* (SMc00975) (ref. 51)	Proline, histidine, lysine

^*^Locus tag identifiers for each protein are provided.
