# Supplementary material for: A direct-sensing galactose chemoreceptor recently evolved in invasive strains of Campylobacter jejuni
Source: Nat Commun. 2016 Oct 20;7:13206. doi: 10.1038/ncomms13206 (PMC5080441; doi:10.1038/ncomms13206)
Supplement: Supplementary Information — Supplementary Figures 1 - 4, Supplementary Tables 1 - 4 and Supplementary References [file ncomms13206-s1.pdf]

Supplementary Information

Supplementary Figure 1

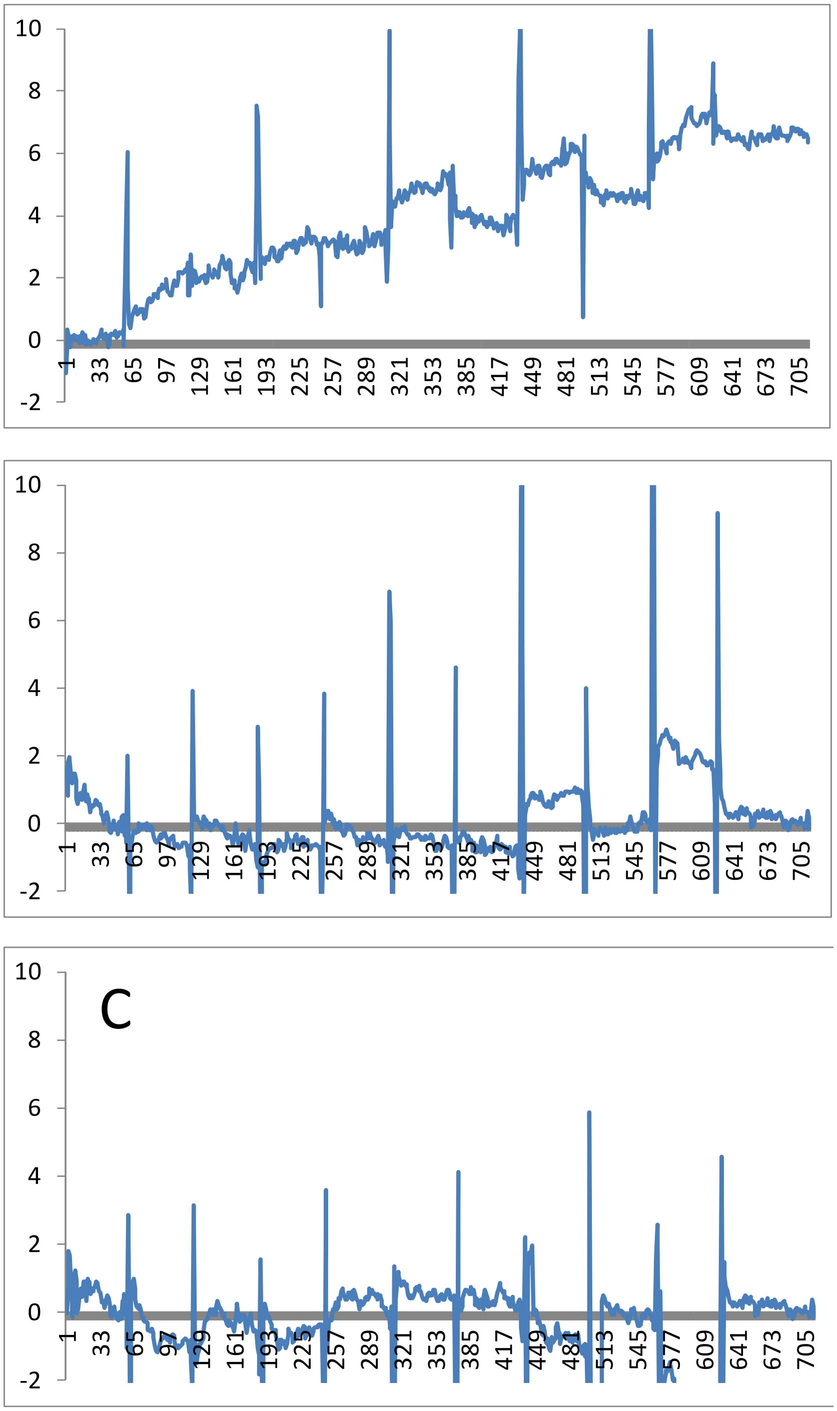

Suplementary Figure 1 SPR sensorgrams of interactions with CcrG. **(A)** Interaction between CcrG and Galactose. **(B)** Interaction between CcrG and Glucose. **(C)** Interaction between CcrG and Ribose.

Supplementary Figure 2

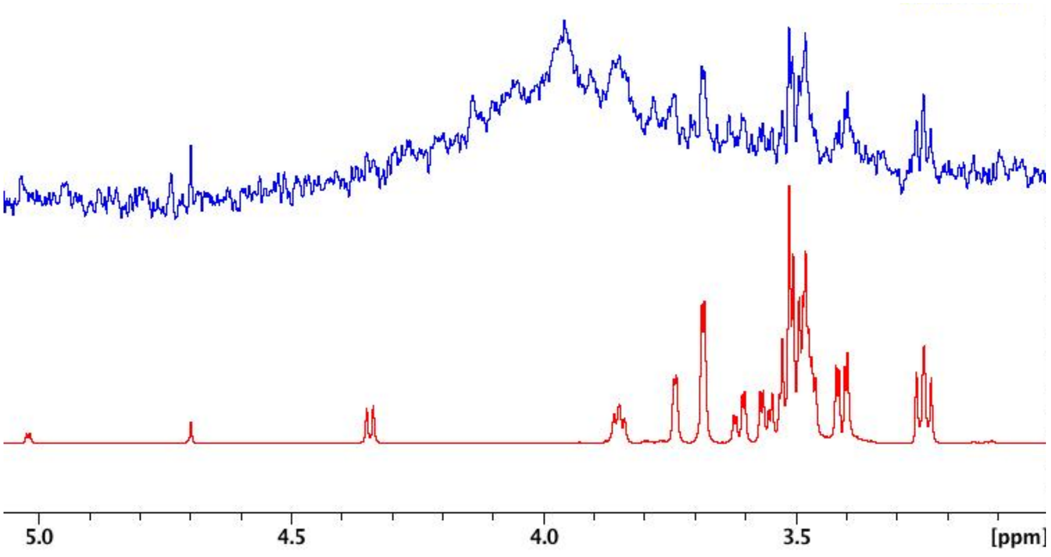

Supplementary Figure 2. ^{1}H NMR spectrum of galactose (bottom spectrum, 3.0 – 5.1 ppm, 600 MHz, 278 K) and the ^{1}H STD NMR spectrum of tlp11 and galactose (top spectrum, 3.0 – 5.1 ppm, 600 MHz, 278 K). Control STD NMR experiments were performed using an identical experimental setup and the same ligand concentration but in the absence of the protein.

Supplementary Figure 3

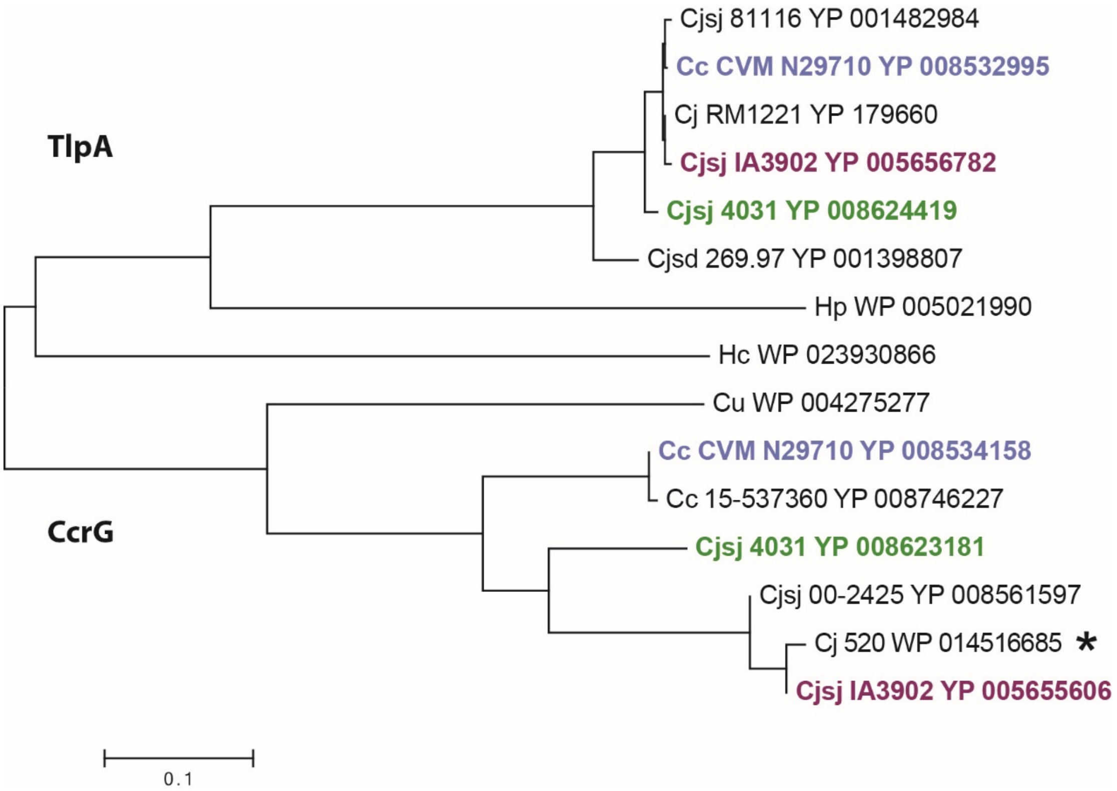

Supplementary Figure 3. Relationships between CcrG orthologs and their closest homologs, TlpA. The maximum likelihood tree was constructed from multiple sequence alignment of protein sequences. CcrG from *C. jejuni* 520 used throughout this study is marked by an asterisk. CcrG and TlpA groups are clearly separated by the longest branch. Sequence identifiers include the abbreviated species name followed by the strain name and NCBI accession number. Sequences from the same strains are shown in the same color. Strain abbreviations: Cc, *Campylobacter coli*; Cj, *Campylobacter jejuni*; Cjsd, *Campylobacter jejuni* subsp. *doylei*; Cjsj, *Campylobacter jejuni* subsp. *jejuni*; Cu, *Campylobacter upsaliensis*; Hc, *Helicobacter canis*; Hp, *Helicobacter pylori*

Supplementary Figure 4

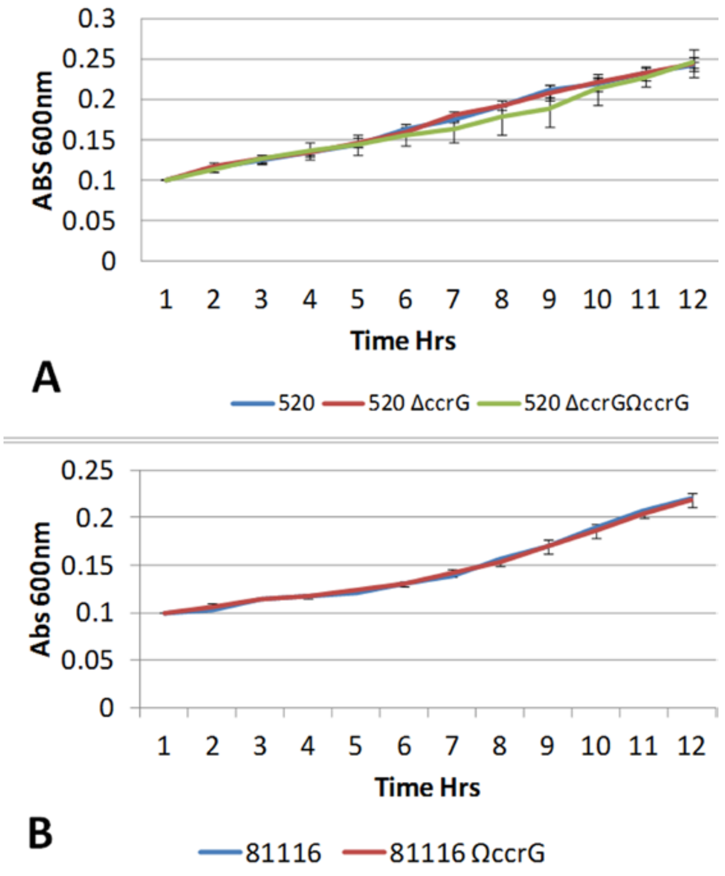

Supplementary Figure S4. Growth rate curves. **A**, *C. jejuni* 520 (wild-type), 520ΔccrG::Km^R, and 520ΔccrGΩccrG; **B**, *C. jejuni* 811116 (wild type) and 81116 ΩccrG. No significant differences were observed between the wild-type strains and their isogenic mutants between three replicate (N=3) growth curves and standard deviation errors are shown as bars above the mean (p>0.05; T-test).

Supplementary Tables

Supplementary Table 1. Distribution of Tlp11 in invasive and non-invasive strains of *C. jejuni*, isolated from human and avian sources.

Human isolates	Invasive status *	CcrG (Tlp11)	Chicken isolates	Invasive status	CcrG (Tlp11)
FF3	+++	-	007	+++	-
705	++	-	017	++	-
FF13	++++	+	115	-	-
FF18	+/-	-	133	++	-
FF34	-	-	301	++	-
108a	+	-	311	+	-
224	+++	-	331	-	-
351	+	-	405	-	-
354	++	-	412	++	-
81116	+	-	413	+	-
435	++	-	415	++	-
440	+/-	-	421	+++	-
464	+/-	-	423	+++	+
520	++++	+	315	-	-
835	++	-	506	-	-
860	+	+	802	++	-
872	+++	-	904	-	-
873	-	-	913	+	-
884	++	+	933	++	-
886	+++	-	952	++	-
887	-	-	7001	-	-
957	-	-	8001	-	-
NCTC11168	+++	-			
NCTC11351	+	-			

*+ represents invasive cell count of 10^1; ++ represents invasive cell count of 10^2; +++ represents invasive cell count of 10^3; ++++ represents invasive cell count of 10^4
The cell counts represent an average of three replicate experiments (N=3).

Supplementary Table 2. Plasmids and bacterial strains used in this study

Strain or plasmid	Genotype or description	Antibiotic resistance	Source or reference
E. coli HB101	Cloning strain		[1]
E. coli DH5α	Cloning strain		[2]
Salmonella typhimurium	Wild type		[3]
C. jejuni 520, 34 and all strains in Table S1	Wild type		Griffith/RMIT* culture collection
520 *ccrG* mutant	520Δ*ccrG*::Km^R	Km	This study
520 *ccrG* complemented mutant	520Δ*ccrG*::Km^RΩ*ccrG*::Cat (Abbreviated to 520Δ*ccrG* Ω*ccrG*)	Km, Cm	This study
520 *ccrG* incorporated into genome of 81116	81116Ω*ccrG*	Km	This study
520 *ccrG* incorporated into genome of FF34	FF34Ω*ccrG*	Km	This study
pBR322	Cloning vector	Amp	[4]
Saccharomyces cerevisae AH109	Yeast two-hybrid strain		Clontech
pGEM®-T-Easy (pGE)	Cloning vector	Amp	Promega
pMW10	Vector with *kanamycin* cassette from *C. coli*	Amp, Km	[5]
pBF6A	pBluscriptΩ*C.jejuni::flaA::flaB::*$Km^R$$Amp^R$;	Amp, Km	Fry, B**, unpublished
pGU0203	pGEM with *ccrG* 520 and flanking sequence inserted into *Bgl*II site	Amp	This study
pGU0204	pGU0203 with deletion in *ccrG* replaced by Km^R cassette : Δ*ccrGP*::Km^R	Amp, Km	This study
pGU0205	pBF6A with 520 *ccrG* incorporated into genome of 81116	Amp, Km	This study
pGADT7	GAL-4 activation domain expression vector	Amp	Clontech

pGBKT7	GAL-4 DNA binding domain expression vector	Km	Clontech
pGADT7-T	Allows expression of the SV40 large T-antigen as a fusion to the GAL-4 AD	Amp	Clontech
pGBKT7-53	Allows expression of the murine p53 protein as a fusion to the GAL-4 DNA-BD, used as positive control when co- transformed with pGADT7-T	Km	Clontech
pGBKT7-Lam	Allows expression of the human lamin C protein as a fusion to the GAL-4 DNA-BD, used as a negative control when co-transformed with pGADT7-T	Km	Clontech
pADCcrG^{sig}	DNA sequence encoding residues 562 – 707 of CcrG cloned into pGADT7	Amp	This study
pBKCcrG^{sig}	DNA sequence encoding residues 562 – 707 of CcrG cloned into pGBKT7	Km	This study
pBKcheW	*cheW* cloned into pGBKT7	Km	[6]
pBKcheV	*cheV* cloned into pGBKT7	Km	[6]
pBKcheV^{dW}	DNA sequence encoding the CheW-like domain of CheV cloned into pGBKT7	Km	[6]
pBKcheY	*cheY* cloned into pGBKT7	Km	[6]
pADcheW	*cheW* cloned into pGADT7	Amp	[6]
pADcheV	*cheV* cloned into pGADT7	Amp	[6]
pADcheV^{dW}	DNA sequence encoding the CheW-like domain of CheV cloned into pGADT7	Amp	[6]
pADcheY	*cheY* cloned into pGADT7	Amp	[6]
pBKcheA	*cheA* cloned into pGBKT7	Km	[6]

[*] Griffith University and RMIT (Royal Melbourne Institute of Technology) University culture collections. [**] Benjamin Fry, RMIT University, Melbourne, Vic 3000, Australia**.**

Supplementary Table 3. Glycans printed on glycan array

Identifier*	Name	Structure
	**Terminal Galactose**	
1A	Lacto-*N*-Biose I	Galβ1-3Glc*N*Ac
1B	N-Acetyllactosamine	Galβ1-4Glc*N*Ac
1C	β1-4galactosyl-galactose	Galβ1-4Gal
1D	β1-6galactosyl-*N*-acetylglucosamine	Galβ1-6Glc*N*Ac
1E	β1-3galactosyl-*N*-acetylgalactosamine	Galβ1-3Gal*N*Ac
1F	AsialoG_{M1}	Galβ1-3Gal*N*Acβ1-4Galβ1-4Glc
1G	Lacto-*N*-tetrose	Galβ1-3Glc*N*Acβ1-3Galβ1-4Glc
1H	Lacto-*N*-neotetrose	Galβ1-4Glc*N*Acβ1-3Galβ1-4Glc
1I	Lacto-*N*-neohexose	Galβ1-4Glc*N*Acβ1-6(Galβ1-4Glc*N*Acβ1-3)Galβ1-4Glc
1J	Lacto-*N*-hexose	Galβ1-4Glc*N*Acβ1-6(Galβ1-3Glc*N*Acβ1-3)Galβ1-4Glc
1K	Globotriose	Galα1-4Galβ1-4Glc
1L	Tn Antigen	Gal*N*Acα1-*O*-Ser
1M	Galactosyl-Tn Antigen	Galβ1-3Gal*N*Acα1-*O*-Ser
1N	α1-3 Galactobiose	Galα1-3Gal
1O	Linear B-2 Trisaccharide	Galα1-3Galβ1-4Glc*N*Ac
1P	Linear B-6 Trisaccharide	Galα1-3Galβ1-4Glc
2A	α1-3, β1-4, α1-3 Galactotetrose	Galα1-3Galβ1-4Galα1-3Gal
2B	β1-6Galactobiose	Galβ1-6Gal
2C	Terminal disaccharide of globotriose	Gal*N*Acβ1-3Gal
2D	Receptor for pili of *P. aeruginosa*	Gal*N*Acβ1-4Gal

2E	P1 Antigen	Galα1-4Galβ1-4Glc*N*Ac
2F	α-D-*N*-acetylgalactosaminyl-1-3Gal-β1-4Glc	Gal*N*Acα1-3Galβ1-4Glc
2G	iso-Lacto-*N*-octose	Galβ1-3Glc*N*Acβ1-3Galβ1-4Glc*N*Acβ1-6(Galβ1-3Glc*N*Acβ1-3)Galβ1-4Glc
2H	*para*-Lacto-*N*-hexose	Galβ1-3Glc*N*Acβ1-3Galβ1-4Glc*N*Acβ1-3Galβ1-4Glc
	**Terminal *N*`Acetyl glucosamine**	
4A	*N*,*N'*-Diacetyl chitobiose	Glc*N*Acβ1-4Glc*N*Ac
4B	*N*,*N'*,*N''*-Triacetyl chitotriose	Glc*N*Acβ1-4Glc*N*Acβ1-4Glc*N*Ac
4C	*N*,*N'*,*N''*,*N'''*-Tetraacetyl chitotetrose	Glc*N*Acβ1-4Glc*N*Acβ1-4Glc*N*Acβ1-4Glc*N*Ac
4D	*N*,*N'*,*N''*,*N'''*,*N''''*,*N'''''*-Hexaacetyl chitohexose	Glc*N*Acβ1-4Glc*N*Acβ1-4Glc*N*Acβ1-4Glc*N*Acβ1-4Glc*N*Acβ1-4Glc*N*Ac
4E	Bacterial cell wall muramyl discaccharide	Glc*N*Acβ1-4Mur*N*Ac
	**Mannose containing structures**	
5A	β1-2-*N*-Acetylglucosamine-mannose	Glc*N*Acβ1-2Man
5B	Bianntennary *N*-linked core pentasaccharide	Glc*N*Acβ1-2Manα1-6(Glc*N*Acβ1-2Manα1-3)Man
5C	α1-2-Mannobiose	Manα1-2Man
5D	α1-3-Mannobiose	Manα1-3Man
5E	α1-4-Mannobiose	Manα1-4Man
5F	α1-6-Mannobiose	Manα1-6Man
5G	α1-3, α1-6-Mannobiose	Manα1-6(Manα1-3)Man
5H	α1-3, α1-3, α1-6-Mannopentaose	Manα1-6(Manα1-3)Manα1-6(Manα1-3)Man
	**Fucosylated structures**	
7A	Lacto-*N*-fucopentose I	Fucα1-2Galβ1-3Glc*N*Acβ1-3Galβ1-4Glc

7B	Lacto-*N*-fucopentose II	Galβ1-3(Fucα1-4)Glc*N*Acβ1-3Galβ1-4Glc
7C	Lacto-*N*-fucopentose III	Galβ1-4(Fucα1-3)Glc*N*Acβ1-3Galβ1-4Glc
7D	Lacto-*N*-difucohexose I	Fucα1-2Galβ1-3(Fucα1-4)Glc*N*Acβ1-3Galβ1-4Glc
7E	Lacto-*N*-difucohexose II	Galβ1-3(Fucα1-4)Glc*N*Acβ1-3Galβ1-4(Fucα1-3)Glc
7F	H-disaccharide	Fucα1-2Gal
7G	2'-Fucosyllactose	Fucα1-2Galβ1-4Glc
7H	3'-Fucosyllactose	Galβ1-4(Fucα1-3)Glc
7I	Lewis^x	Galβ1-4(Fucα1-3)Glc*N*Ac
7J	Lewis^a	Galβ1-3(Fucα1-4)Glc*N*Ac
7K	Blood Group A-trisaccharide	Gal*N*Acα1-3(Fucα1-2)Gal
7L	Lactodifucotetrose	Fucα1-2Galβ1-4(Fucα1-3)Glc
7M	Blood Group B-Trisaccharide	Galβ1-3(Fucα1-2)Gal
7N	Lewis^y	Fucα1-2Galβ1-4(Fucα1-3)Glc*N*Ac
7O	Blood Group H Type II Trisaccharide	Fucα1-2Galβ1-3Glc*N*Ac
7P	Lewisb tetrasaccharide	Fucα1-2Galβ1-3(Fucα1-4)Glc*N*Ac
8A	Sulpho Lewis^a	SO_3-3Galβ1-3(Fucα1-4)Glc*N*Ac
8B	Sulpho Lewis^x	SO_3-3Galβ1-4(Fucα1-3)Glc*N*Ac
8C	Monofucosyl-para-Lacto-*N*-hexose IV	Galβ1-3Glc*N*Acβ1-3Galβ1-4(Fucα1-3)Glc*N*Acβ1-3Galβ1-4Glc
8D	Monofucosyllacto-*N*-hexose III	Galβ1-4(Fucα1-3)Glc*N*Acβ1-6(Galβ1-3Glc*N*Acβ1-3)Galβ1-4Glc
8E	Difucosyllacto-*N*-hexose	Galβ1-4(Fucα1-3)Glc*N*Acβ1-6(Fucα1-2Galβ1-3Glc*N*Acβ1-3)Galβ1-4Glc
8F	Trifucosyllacto-*N*-hexose	Galβ1-4(Fucα1-3)Glc*N*Acβ1-6(Fucα1-2Galβ1-3(Fucα1-4)Glc*N*Acβ1-3)Galβ1-4Glc

8G	Lacto-*N*-fucopentaose VI	Galβ1-4GlcNAcβ1-3Galβ1-4(Fucα1-3)Glc
8H	Lacto-*N*-neodifucohexaose I	Fucα1-2Galβ1-4(Fucα1-3)GlcNAcβ1-3Galβ1-4Glc
8I	Lacto-*N*-neodifucohexaose II	Fucα1-3Galβ1-4GlcNAcb1-3Galβ1-4(Fucα1-3)Glc
8J	Trifucosyllacto-*N*-neoteraose I	Fucα1-2Galβ1-4(Fucα1-3)GlcNAcβ1-3(Fucα1-2)Galβ1-4Glc
8K	Monofucosyllacto-*N*-neohexaose I	Galβ1-4(Fucα1-3)GlcNAcβ1-6(Galβ1-4GlcNAcβ1-3)Galβ1-4Glc
8L	Difucosyllacto-*N*-neohexaose I	Galβ1-4(Fucα1-3)GlcNAcβ1-6(Galβ1-4(Fucα1-3)GlcNAcβ1-3)Galβ1-4Glc
8M	Difucosyllacto-*N*-neohexaose II	Fucα1-2Galβ1-4(Fucα1-3)GlcNAcβ1-6(Galβ1-4GlcNAcβ1-3)Galβ1-4Glc
8N	Monofucosyl(1-3)-iso-lacto-*N*-octaose	Galβ1-3GlcNAcβ1-3Galβ1-4(Fucα1-3)GlcNAcβ1-6(Galβ1-3GlcNAcβ1-3)Galβ1-4Glc
8O	Trifucosyl(1-2,1-2,1-3)-iso-lacto-N-octaose	Fucα1-2Galβ1-3GlcNAcβ1-3Galβ1-4(Fucα1-3)GlcNAcβ1-6(Galβ1-3GlcNAcβ1-3)Galβ1-4Glc
8P	Blood Group A Tetrasaccharide	GalNAcα1-3(Fucα1-2)Galβ1-4Glc
9A	Blood Group B pentasaccharide	Galα1-3(Fucα1-2)Galβ1-4(Fucα1-3)Glc
	**Neu5Ac containing structures**	
10A	Sialyl Lewis^a	Neu5Acα2-3Galβ1-3(Fucα1-4)Glc*N*Ac
10B	Sialyl Lewis^x	Neu5Acα2-3Galβ1-4(Fucα1-3)Glc*N*Ac
10C	Sialyllacto-*N*-tetrose a	Neu5Acα2-3Galβ1-3Glc*N*Acβ1-3Galβ1-4Glc
10D	Monosialyl, monofucosyllacto-*N*-neohexose	Galβ1-4(Fucα1-3)Glc*N*Acβ1-6(Neu5Acα2-6Galβ1-4Glc*N*Acβ1-3)Galβ1-4Glc

10K	2,3'-Sialyllactosamine	Neu5Acα2-3Galβ1-4Glc*N*Ac
10L	2,6'-Sialyllactosamine	Neu5Acα2-6Galβ1-4Glc*N*Ac
10M	LS-Tetrasaccharide a	Neu5Acα2-3Galβ1-3GlcNAcβ1-3Galβ1-4Glc
10N	LS-Tetrasaccharide b	Galβ1-3(Neu5Acα2-6)Glc*N*Acβ1-3Galβ1-4Glc
10O	LS-Tetrasaccharide c	Neu5Acα2-6Galβ1-4Glc*N*Acβ1-3Galβ1-4Glc
10P	Disialyllacto-*N*-tetrose	Neu5Acα2-3Galβ1-3(Neu5Acα2-6)Glc*N*Acβ1-3Galβ1-4Glc
11A	2,3'-Sialyllactose	Neu5Acα2-3Galβ1-4Glc
11B	2,6'-Sialyllactose	Neu5Acα2-6Galβ1-4Glc
11C	Colominic acid	(Neu5Acα2-8Neu5Ac)n (n<50)
11D	Biantennary 2,6-sialylated-*N*-glycan-Asn	Neu5Acα2-6Galβ1-4Glc*N*Acβ1-2Manα1-6(Neu5Acα2-6Galβ1-4Glc*N*Acβ1-2Manα1-6)Manβ1-4Glc*N*Acβ1-4Glc*N*Ac-Asn
	**Carageenan and Glycoaminoglycans (GAGS**	
12A	Neocarratetrose-41, 3-di-*O*-sulphate (Na^+)	$C_{24}H_{36}O_{25}S_2Na_2$ (Mixed anomers. Tetrasaccharide of regular κ -carrageenan)
12B	Neocarratetrose-41-*O*-sulphate (Na^+)	$C_{24}H_{37}O_{22}SNa$ (Mixed anomers. Derived from C1003 by removal of the non-reducing terminal 4-sulphate)
12C	Neocarrahexose-24,41, 3, 5-tetra-*O*-sulphate (Na^+)	$C_{36}H_{52}O_{40}S_4Na_4$ (Mixed anomers. A hybrid sequence comprising carrageenan disaccharides in the order κ–ι–κ, derived from the carrageenan from *Chondrus crispus*)
12D	Neocarrahexose-41, 3, 5-tri-*O*-sulphate (Na^+)	$C_{36}H_{53}O_{37}S_3Na_3$ (Mixed anomers. Hexasaccharide of regular κ-carrageenan)
12E	Neocarraoctose-41, 3, 5, 7-tetra-*O*-sulphate (Na^+)	$C_{48}H_{70}O_{49}S_4Na_4$ (Mixed anomers. Octasaccharide of regular κ-carrageenan)

12F	Neocarradecose-41, 3, 5, 7, 9-penta-*O*-sulphate (Na^+)	$C_{60}H_{87}O_{61}S_5Na_5$ (Mixed anomers. Decasaccharide of regular κ-carrageenan)
12G	ΔUA-2S → Glc*N*S-6S Na_4 (I-S)	$C_{12}H_{15}NO_{19}S_3Na_4$ (Predominant disaccharide produced from heparin by heparinase I and II)
12H	ΔUA → Gluc*N*S-6S Na_3 (II-S)	$C_{12}H_{16}NO_{16}S_2Na_3$ (Produced from heparinase II digestion of heparin and heparin sulphate)
12I	ΔUA → 2S-Glc*N*S Na_3 (III-S)	$C_{12}H_{16}NO_{16}S_2Na_3$ (Produced from heparin by digestion with heparinase I and II)
12J	ΔUA → 2S-Glc*N*Ac-6S Na_3 (I-A)	$C_{14}H_{18}NO_{17}S_2Na_3$ (Minor component produced from heparin by heparinase II)
12K	ΔUA → Glc*N*Ac-6S Na_2 (II-A)	$C_{14}H_{19}NO_{14}SNa_2$ (Product of the action of heparinases II and III on heparin and heparan sulphate)
12L	ΔUA → 2S-Glc*N*Ac Na_2 (III-A)	$C_{14}H_{19}NO_{14}SNa_2$ (Minor product of the action of heparinase II on heparin)
12M	ΔUA → Glc*N*Ac Na (IV-A)	$C_{14}H_{20}NO_{11}Na$ (Produced from heparin sulphate by digestion With heparinase III)
12N	ΔUA → Gal*N*Ac-4S Na_2 (ΔDi-4S)	$C_{14}H_{19}NO_{14}SNa_2$ (Produced from various chondroitin sulphates By the action of chondroitinases ABC, B and AC-1)
12O	ΔUA → Gal*N*Ac-6S Na2 (ΔDi-6S)	$C_{14}H_{19}NO_{14}SNa_2$ (Produced from various chondroitin sulphates By the action of chondroitinases ABC, AC-1 and C)
12P	ΔUA → Gal*N*Ac-4S,6S Na_3 (ΔDi-disE)	$C_{14}H_{18}NO_{17}S_2Na_3$ (Produced from various chondroitin sulphates By the action of chondroitinases ABC, B and AC-1)
13A	ΔUA → 2S-Gal*N*Ac-4S Na_2 (ΔDi-disB)	$C_{14}H_{18}NO_{17}S_2Na_3$ (Produced from various chondroitin sulphates by action of chondroitinase ABC and/or B. Most

		typically from chondroitin sulphate B (dermatan sulphate))
13B	ΔUA → 2S-Gal*N*Ac-6S Na_3 (ΔDi-disD)	$C_{14}H_{18}NO_{17}S_2Na_3$ (Produced from various chondroitin sulphates by the action of chondroitinase ABC)
13C	ΔUA → 2S-Gal*N*Ac-4S-6S Na_4 (ΔDi-tisS)	$C_{14}H_{17}NO_{20}S_3Na_4$ (Produced as a minor component by the action of chondroitinase ABC on various chondroitin sulphates, particularly B)
13D	ΔUA → 2S-Gal*N*Ac-6S Na_2 (ΔDi-UA2S)	$C_{14}H_{19}NO_{14}SNa_2$ (Produced as a minor component from various chondroitin sulphates by the action of chondroitinase ABC)
13E	ΔUA → Glc*N*Ac Na (ΔDi-HA)	$C_{14}H_{20}NO_{11}Na$ (The only unsaturated disaccharide produced from hyaluronic acid by the action of chondroitinase ABC or AC-1)
13F	Hyaluronan fragments (4mer)	(GlcAβ1-3Glc*N*Acβ1-4)n (n=4)
13G	Hyaluronan fragments (8mer)	(GlcAβ1-3Glc*N*Acβ1-4)n (n=8)
13H	Hyaluronan fragments (10mer)	(GlcAβ1-3Glc*N*Acβ1-4)n (n=10)
13I	Hyaluronan fragments (12mer)	(GlcAβ1-3Glc*N*Acβ1-4)n (n=12)
13J	Heparin	(GlcA/IdoAα/β1-4Glc*N*Acα1-4)n (n=200)
13K	Chondroitin sulfate	(GlcA/IdoAβ1-3(±4/6S)Gal*N*Acβ1-4)n (n<250)
13L	Dermatan sulfate	((±2S)GlcA/IdoAα/b1-3(±4S)Gal*N*Acβ1-4)n (n<250)
13M	Chondroitin 6-Sulfate	(GlcA/IdoAβ1-3(±6S)Gal*N*Acβ1-4)n (n<250)
13N	HA - 4	(GlcAβ1-3Glc*N*Acβ1-4)n (n=4)
130	HA - 6	(GlcAβ1-3Glc*N*Acβ1-4)n (n=6)
13P	HA - 8	(GlcAβ1-3Glc*N*Acβ1-4)n (n=8)
14A	HA 10	(GlcAβ1-3Glc*N*Acβ1-4)n (n=10)

14B	HA-12	(GlcAβ1-3Glc*N*Acβ1-4)n (n=12)
14C	HA-14	(GlcAβ1-3Glc*N*Acβ1-4)n (n=14)
14D	HA-16	(GlcAβ1-3Glc*N*Acβ1-4)n (n=16)
14E	HA 30000 Da	(GlcAβ1-3Glc*N*Acβ1-4)n
14F	HA 107000 Da	(GlcAβ1-3Glc*N*Acβ1-4)n
14G	HA 190000 Da	(GlcAβ1-3Glc*N*Acβ1-4)n
14H	HA 220000 Da	(GlcAβ1-3Glc*N*Acβ1-4)n
14I	HA 1600000 Da	(GlcAβ1-3Glc*N*Acβ1-4)n
14J	Heparin sulfate	(GlcA/IdoAα/ IdoASα/β1-4Glc*N*Ac/GlcNS/Glc*N*Ac6Sα1-4)n
14K	b1-3Glucan	(Glcβ1-4Glc)n

*Identifier: Plate location for printing identification.

Supplementary Table 4. Analysis of protein interactions with Tlp11^{sig} using the yeast two-hybrid system

**AD-CcrG^{sig} with:**		**BD-Tlp11^{sig} with:**	
BD-CcrG^{sig}	+++	AD-CcrG^{sig}	+++
BD-CheW	0	AD-CheW	+
BD-CheV	++	AD-CheV	++
BD-CheV^{dW}	++	AD-CheV^{dW}	0
BD-CheY	0	AD-CheY	0

Testing for interactions between CcrG^{sig} fused to the GAL-4 activation domain (AD- CcrG^{sig}) and GAL-4 DNA binding domain (BD- CcrG^{sig}) with selected chemotaxis proteins and individual domains fused to the GAL-4 DNA binding domain (BD) and GAL-4 activation domain (AD) respectively (3 replicate experiments, n=3). Co-transformation of AD-CheW and BD-CheA was used as a positive control when testing for protein-protein interactions, with a result of +++.
+++: cream, dense growth of > 75% of co-transformants observed on high stringency media and intermediate stringency media.
++: cream – pink moderate growth for ~75% or more of co-transformants observed on high stringency media and intermediate stringency media.
+: cream – pink light growth for ~50% or more of co-transformants observed on high stringency media and/or intermediate stringency media.
0: no growth of co-transformants observed.

The reciprocal interaction between BD-CcrG^{sig} and AD-CheV^{dW} could not be seen and may have gone undetected due to the presence of 1mM 3-AT in the intermediate stringency media used to assess interactions with BD-CcrG^{sig}. This was necessary as autonomous activation of reporter gene expression was observed with BD-CcrG^{sig}. The CheY protein was included in the two-hybrid analyses as a negative control, while the AD-CheW and BD-CheA fusion proteins were included as a positive control producing a strong interaction.

Reference list

1 Boyer, H. W. & Roulland-Dussoix, D. A complementation analysis of the restriction and modification of DNA in Escherichia coli. *J Mol Biol* **41**, 459-472 (1969).

2 Hanahan, D. Studies on transformation of *Escherichia coli* with plasmids. *J Mol Biol* **166**, 557-580 (1983).

3 Wilkinson, R. G., Gemski, P. J. & Stocker, B. A. Non-smooth mutants of *Salmonella typhimurium:* differentiation by phage sensitivity and genetic mapping. *J Gen Microbiol.* **70**, 527-554 (1972).

4 Bolivar, F. *et al.* Construction and characterization of new cloning vehicles. II. A multipurpose cloning system. *Gene* **2**, 95-113 (1977).

5 Wosten, M. M. S. M., Boeve, M., Koot, M. G. A., van Nuenen, A. C. & van der Zeijst, B. A. M. Identification of *Campylobacter jejuni* Promoter Sequences. *Journal of Bacteriology* **180**, 594-599 (1998).

6 Hartley-Tassell, L. E. *et al.* Identification and characterization of the aspartate chemosensory receptor of *Campylobacter jejuni*. *Mol Microbiol* **75**, 710-730(2010).
